# The Glycosyltransferase Repertoire of the Spikemoss *Selaginella moellendorffii* and a Comparative Study of Its Cell Wall

**DOI:** 10.1371/journal.pone.0035846

**Published:** 2012-05-02

**Authors:** Jesper Harholt, Iben Sørensen, Jonatan Fangel, Alison Roberts, William G. T. Willats, Henrik Vibe Scheller, Bent Larsen Petersen, Jo Ann Banks, Peter Ulvskov

**Affiliations:** 1 Department of Plant Biology and Biotechnology, VKR Research Centre “Pro-Active Plants,” University of Copenhagen, Frederiksberg, Denmark; 2 Department of Plant Biology, Cornell University, Ithaca, New York, United States of America; 3 Department of Biological Sciences, University of Rhode Island, Kingston, Rhode Island, United States of America; 4 Feedstocks Division, Joint Bioenergy Institute, Emeryville, California, United States of America; 5 Physical Biosciences Division, Lawrence Berkeley National Laboratory, Berkeley, California, United States of America; 6 Department of Plant and Microbial Biology, University of California, Berkeley, California, United States of America; 7 Department of Botany and Plant Pathology, Purdue University, West Lafayette, Indiana, United States of America; University of Melbourne, Australia

## Abstract

Spike mosses are among the most basal vascular plants, and one species, *Selaginella moellendorffii*, was recently selected for full genome sequencing by the Joint Genome Institute (JGI). Glycosyltransferases (GTs) are involved in many aspects of a plant life, including cell wall biosynthesis, protein glycosylation, primary and secondary metabolism. Here, we present a comparative study of the *S. moellendorffii* genome across 92 GT families and an additional family (DUF266) likely to include GTs. The study encompasses the moss *Physcomitrella patens*, a non-vascular land plant, while rice and Arabidopsis represent commelinid and non-commelinid seed plants. Analysis of the subset of GT-families particularly relevant to cell wall polysaccharide biosynthesis was complemented by a detailed analysis of *S. moellendorffii* cell walls. The *S. moellendorffii* cell wall contains many of the same components as seed plant cell walls, but appears to differ somewhat in its detailed architecture. The *S. moellendorffii* genome encodes fewer GTs (287 GTs including DUF266s) than the reference genomes. In a few families, notably GT51 and GT78, *S. moellendorffii* GTs have no higher plant orthologs, but in most families *S. moellendorffii* GTs have clear orthologies with Arabidopsis and rice. A gene naming convention of GTs is proposed which takes orthologies and GT-family membership into account. The evolutionary significance of apparently modern and ancient traits in *S. moellendorffii* is discussed, as is its use as a reference organism for functional annotation of GTs.

## Introduction

*Selaginella moellendorffii* is an extant member of the lycopsid lineage, which diverged from the Euphyllophyta (ferns and seed plants) ca. 400 Myr ago [1]. *S. moellendorffii* was selected for sequencing by the Joint Genome Institute (JGI) because of its important evolutionary position among land plants and its small genome size [2], [3]. Unlike bryophytes, which have a dominant gametophyte phase, the sporophyte is dominant phase of the lycopsid life cycle. Lycopsids are further set apart from the mosses by the presence of a true vascular system for water and assimilate transport.

The moss *Physcomitrella patens* is the only non-vascular terrestrial plant and the only other non-angiosperm plant that has had its genome sequenced, and it is thus particularly important to the comparative genomic study of *S. moellendorffii* [4]. Bryophytes represent the oldest extant terrestrial plants and have diverged markedly from their Charophycean algal ancestors. Biochemical studies of basal plants and Charophycean algae indicate that the widespread utilization of some cell wall features such as rhamnogalacturonan II (RG-II) and lignin, or at least uncondensed monolignols, occurred concurrently with the colonization of land [5]. Xylan has been found throughout the green plant lineage while the common ancestor of all plants with xyloglucan (XyG) probably was a Charophycean alga [6]. XyG fine structure displays variations within vascular plants that appear to reflect taxonomy [7].

Presumably, specific elaborations in cell wall architecture are correlated with the particular lifestyles of individual plant species and in some cases mark major evolutionary transitions. One of the most important innovations following colonization of dry land was the tracheary element from which the term tracheophyte (vascular plant) is derived. Tracheary elements are hollow, rigid cells, which have undergone cell death and are specialized for water transport. They first appear in the fossil record during the late Silurian period [8] and were important for the radiation of vascular plants during the Devonian period [9].

The complex polysaccharides of cell walls are synthesized by the coordinated activity of a large set of carbohydrate active enzymes, including glycosyltransferases (GTs) that catalyze the formation of glycosidic bonds between donor sugars and acceptor molecules and that are the primary focus of this study. The Carbohydrate Active enZyme, or CAZy, database [10] is of increasing importance to cell wall biology and we have used a CAZy-driven approach for analyzing the *S. moellendorffii* genome.

Some clades of putative cell wall biosynthetic enzymes are shared by *P. patens*, *S. moellendorffii* and seed plants whereas others diversified more recently and are restricted to one of the three lineages. These observations lead us to propose that although lycophytes share many cell wall features with the other major clades of land plants, *S. moellendorffii* (as well as *P. patens*) has undergone independent diversification of some gene families and retained ancestral genes that have been lost in seed plants. The evolutionary divergence of GTs and cell wall structure is presumably a reflection of the different adaptive strategies that predominate within the major lineages.

Comparative studies of cell wall biosynthesis proteomes should ideally be paralleled by comparative studies of the corresponding cell wall composition and architecture, in this case the analysis of the cell walls of *S. moellendorffii* compared to those of *P. patens* and of the fully sequenced angiosperms Arabidopsis and rice. In this regard, we present here biochemical and immunolocalisation analyses of *S. moellendorffii* cell walls as a foundation for discussing the cell wall biosynthetic proteome.

## Results and Discussion

### *S. moellendorffii* cell wall composition and architecture

#### Comprehensive Microarray Polymer Profiling

**Figure 1:**
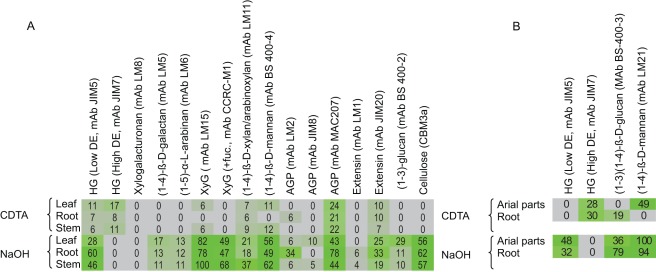
Analysis of polysaccharides in the cell wall of *S. moellendorffii* using the microarray and antibody based CoMPP technique (Moller et al., 2007). Leaves, root and stems (A) and aerial part and roots (B). The heatmap, in which mean spot signals are correlated with colour intensity, shows the relative abundance of cell wall components as extracted using 1,2-diaminocyclohexanetetraacetic acid (CDTA) and NaOH. A low end cut-off value of 5 was used. The same amount of cell wall material (alcohol insoluble residue) was used for each sample. HG, homogalacturonan. XG, xyloglucan. AGP, arabinogalactan protein. This analysis indicates that *S. moellendorffii* has many cell wall polysaccharides in common with higher plants. It is of particular note that in contrast to most other higher plants, HG from *S. moellendorffii* was extracted more readily with NaOH than with CDTA and the occurrence of mixed linkage glucan. However, in common with higher plants but in contrast to *P. patens*, *S. moellendorffii* contained fucosylated XyG.

Cell wall polysaccharides were analysed using the microarray/antibody based Comprehensive Microarray Polymer Profiling or CoMPP technique [11] to obtain semi-quantitative information about the relative abundance of cell wall polysaccharides. CoMPP data for P. patens has previously been reported [11]. CoMPP analysis of S. moellendorffii detected many of the polysaccharides that are typically present in higher plants, including Arabidopsis (Figure 1). We found evidence of glycan epitopes that are typically associated with cellulose, pectins, xyloglucans, xylans, mannans, arabinogalactan-proteins (AGPs) and extensins. The XyG epitopes recognized by LM15 and CCRC-M1 (antibodies that recognize non-fucosylated and fucosylated XyG, respectively) were both present in S. moellendorffii. In contrast, fucosylated XyG epitope recognized by CCRC-M1 was essentially absent from P. patens [11], [12]. The presence of mannan epitopes or polymers in the cell walls of P. patens and the Charaphycean alga Chara corallina [11], [13], [14] in addition to S. moellendorffii indicates that mannose-containing cell wall polymers most likely evolved prior to the colonization of land. As with higher plants, the hemicellulosic and crystalline cellulose epitopes were detected almost exclusively in NaOH extracts. Extensin and AGP epitopes showed similar extraction patterns as observed for Arabidopsis.

Two surprising observations were made in the CoMMP data. Certain pectin epitopes, including homogalacturonan (HG, recognized by mAb JIM5), β-(1,4)-galactan (recognized by mAb LM5) and α-(1,5)-arabinan (recognized by mAb LM6) were extracted effectively only by NaOH and not by CDTA. This is in contrast to most other land plants, including *P. patens*, where these pectic polymers are more readily extracted with CDTA. The reduced CDTA solubility of *S. moellendorffii* HG may indicate a different association between the pectic matrix and other cell wall components. This trend was confirmed in CoMPP analysis of another *Selaginella* species, *S. kraussiana* (data not shown). Moreover, a related pattern was observed in another basal land plant, the horsetail *Equisetum arvense*, where the JIM5 epitope in certain tissues was not completely extractable with CDTA but in the subsequent NaOH treatment (∼20% compared to CDTA) and the LM5 and LM6 epitopes were extracted in highest amounts with the strong base (data not shown)[15].

**Figure 2:**
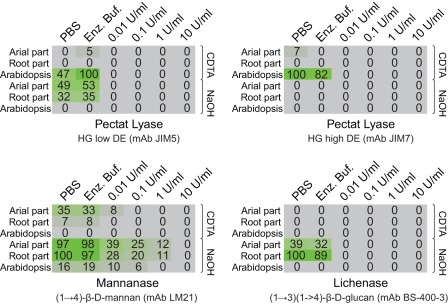
Confirmation that the epitope recognized by homogalacturonan, mixed linkage glucan and mannan specific antibodies are susceptible to enzymatic degradation by enzymes specific for the given polymer. CoMMP arrays were treated with PBS, buffer or increasing amount of enzyme to investigate if the epitope in question could be abolished. In all four cases were the epitope recognized by the antibody susceptible to enzymatic degradation, confirming that the epitope is found in the proposed polymer.

The discovery of ), β-(1,3)(1,4)-glucan (‘mixed linkage glucan’ or MLG) epitopes (as recognized by the anti-MLG monoclonal antibody BS-400-3) was also surprising. MLG has been observed outside the Poales in *Equisetum arvense* but, this is to the author's knowledge, the first report of mixed linkage glucan in the lycophytes [15]. In order to confirm the presence of MLG, arrays of *S. moellendorffii* cell wall material were treated prior to BS-400-3 labelling with lichenase, an enzyme that specifically cleaves MLG. This treatment resulted in the complete abolition of BS-400-3 binding and this strongly suggest that the cell walls of *S. moellendorffii* do indeed contain this polysaccharide. (Figure 2). Similarly were the occurrence of homogalacturonan and mannan confirmed by enzymatic removal of the epitope with polymer specific enzymes (Figure 2).

#### Immunolocalisation of cell wall epitopes

**Figure 3:**
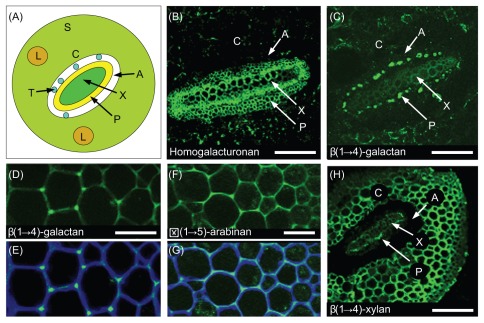
Immunocytochemistry of *S. moellendorffii* stem (A), schematic representation of a section through a *S. moellendorffii* stem showing the location of the cortex (C), sclerenchyma (S), xylem (X), phloem (P), leaf traces (L) and specialized endodermal cells known as trabeculae (T) in the air space (A) separating the stele from the cortex. Indirect immunofluorescence microscopy reveals the location of homogalacturonan (HG, mAb JIM5) (B), β-(1,4)-galactan (mAb LM5) (C), (D) and (E), α-(1,5)-arabinan (mAb LM6) (F) and (G) and β-(1,4)-xylan (mAb LM10) (H). The images in (E) and (G) show counterstaining of the sections shown in (D) and (F) with Calcoflour White that reveals all β-glucan-containing cell walls. In contrast to most higher plants, β-(1,4)-galactan had a highly restricted location in *S. moellendorffii* stems and was located mainly in certain phloem cells (C) and only at the cell junctions of cortical cells (D) and (E). α-(1,5)-arabinan was widely distributed in cortical cell walls, but labelling was restricted to the middle portion of walls rather than throughout their whole width (F) and (G). HG is typically not abundant in the vascular tissues of higher plants but in *S. moellendorffii* HG was located predominantly in the walls of xylem and phloem cells and only present at low levels in other stem tissues. In contrast, labeling of β-(1,4)-xylan was strong in most cell walls apart from those in phloem tissue. Scale bars in (B) and (C)  = 140 μm, in (D) and (F)  = 20 μm, and in (H)  = 200 μm.

A subset of S. moellendorffii cell wall epitopes were immunolocalised in stem sections (Figure 3). The HG epitope recognized by mAb JIM5 was more abundant in the vascular tissues than other stem tissues (Figure 3 A). The binding of the β-(1,4)-galactan mAb LM5 was restricted mostly to the walls of certain phloem cells but in the cortex the LM5 epitope was mainly present in the cell junctions (Figure 3 C, D and E). The α-(1,5)-arabinan epitope recognized by mAb LM6 was widely distributed in cortical cell walls (Figure 3 F and G). In contrast to the pectic epitopes described above, the β-(1,4)-xylan epitope recognized by mAb (LM10) was very widely distributed and labeling was strong in all tissues except the phloem (Figure 3H). Counterstaining with Calcoflour showed that β-glucans were present throughout cell walls and this is consistent with the presence of cellulose (Figure 3 E and G).

#### Cell wall composition analysis

**Figure 4:**
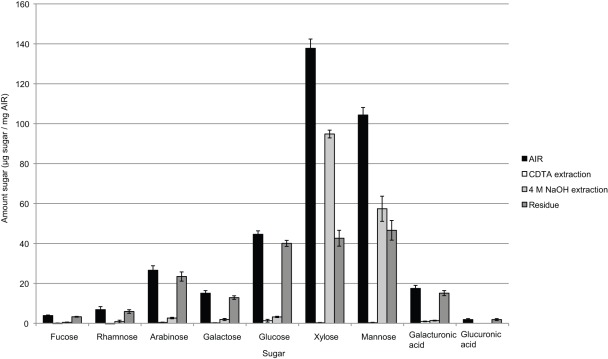
Sequential fractionation of aerial parts of *S. moellendorffii* analyzed with quantitative sugar composition analysis. The recalcitrance to extraction of homogalacturonan as observed in the CoMMP analysis (Figure 1) was confirmed. A general recalcitrance to extraction was observed and only in the cases of xylose and mannose could a majority of the sugars be extracted.

Sugar composition analysis of the aerial parts of S. moellendorffii (Figure 4) revealed that S. moellendorffii cell walls are relatively high in mannose and low in galacturonic acid compared to non-commelinid primary cell walls. The abundance of mannose is consistent with a general trend that the cell walls of basal plant species tend to be mannan-rich, and although the prevalence of this polysaccharide suggests an important role in cell wall physiology our present understanding of this is limited. To investigate the properties of the cell wall further, it was analyzed via sequential extraction using CDTA and 4M NaOH and the sugar composition of the resulting extracts was analysed (Figure 4). In order to explore the recalcitrance to extraction observed in the CoMMP analysis the extraction time was extended to 24 h in CDTA and NaOH extractions. Extraction using CDTA resulted in low amount of sugars extracted. The CDTA contained mainly arabinose, galactose, glucose and galacturonic acid, corresponding to the AGP and HG epitopes found in the CoMMP analysis. The NaOH extraction contained large amounts of xylose and mannose, and in both cases were the majority of xylose and mannose using 4M NaOH. Besides xylose and mannose other sugars were observed, but in low quantities, including the pectinous sugars rhamnose, arabinose, galactose and galacturonic acid and glucose.

Together biochemical and immunochemical data suggest that *S. moellendorffii* contains many of the cell wall polymers that are typical of higher plants including MLG. The cell walls of *S. moellendorffii* are distinct from those of seed plants in the abundance of some polymers, the recalcitrance to extraction, as well as their context both at the cellular and cell wall levels, Both ancestral and derived traits are found intermixed in the *S. moellendorffii* cell wall.

#### The GT repertoire of S. moellendorffii

The global survey of GTs in *S. moellendorffii* was based on a BLAST database built from all GTs in the CAZy database. The entire *S. moellendorffii* and P. patens genomes were run against this database. Due to the quality control measures used (see Materials and Methods) the screen for GT-candidates adopted in this analysis is rather conservative and probably lead to very few false positives, but may have underestimated the true number of GTs in *S. moellendorffii.* Incorrect gene annotations persist in databases long after the mistakes have been discovered and lead to perpetuation of the errors in annotation of other species. False positives are therefore highly undesired while underestimates (false negatives) are less problematic unless conclusions are made on the total number of GTs in a given species.

The *S. moellendorffii* and *P. patens* GTs were classified according to the CAZy nomenclature and named as follows: GT<family number><clade letter><number>. These names are preceded with a species identifier where required, i.e. Sm for *S. moellendorffii.* Other naming conventions have already been implemented for some families. These are respected and *S. moellendorffii* genes are named after their orthologs in these cases. *S. moellendorffii* and *P. patens* protein IDs and gene names are listed by family and clade in Table S1.

**Figure 5:**
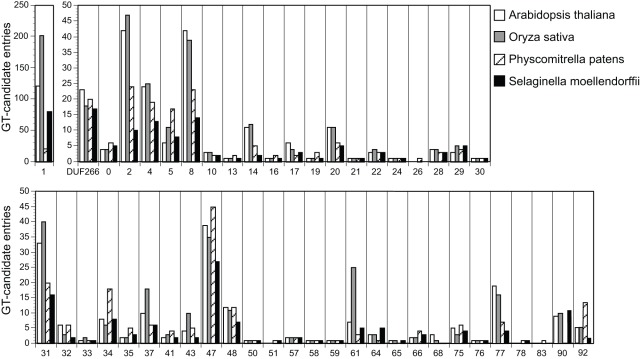
Histogram showing the occurrence of GT-entries across families in the CAZy database and in DUF266-containing genes. *A. thaliana*, white bars; *O. sativa*, grey bars; *P. patens,* hatched bars and *S. moellendorffii*, black bars. GT-numbers on the abscissa are shown in bold for families known or expected to be of particular cell wall relevance. Table S1 lists the JGI protein IDs for both *P. patens* and *S. moellendorffii* and gene names for the latter. Sequences are accessible via the JGI websites and at NCBI.

The histogram in Figure 5 comprises 481, 592, 323 and 287 GTs from Arabidopsis, rice, *P. patens* and *S. moellendorffii*, respectively. *P. patens* has a rather full set of higher plant polysaccharides [15], but a smaller set of GTs might have been expected since the moss lacks several cell types with specialized walls known from vascular plants. However, the *P. patens* genome is larger with 61% more ORFs than *S. moellendorffii*, so the relatively large number reflects that the *P. patens* set of GTs is generally more redundant.

*S. moellendorffii* exhibits both ancestral and derived traits in the identified CAZyome. For example, the genomes of *S. moellendorffi* and *P. patens*, but not seed plants, have members of family GT51, which encode murein synthases involved in cell wall biosynthesis in bacteria, including cyanobacteria from which the *Viridiplantae* chloroplasts arose via an endosymbiotic event (Figure 5). Some of these GTs are penicillin sensitive and interestingly, chloroplast division is sensitive to penicillin in liverworts [16], *P. patens* [17], and *Selaginella nipponica* [18] but not in higher plants. Another striking example is in GT78, containing only two entries namely a mannosylglycerate synthase from *Rhodotermus marinus* (a bacterium) and a homologous partial sequence from *Griffithsia japonicum* (a marine red alga) (Figure 5). Mannosylglycerate is a compatible solute and osmoprotectant [19] produced by many bacteria using either GT78 or, more frequently, GT55 enzymes. GT55 members have not been found within Archaeplastida, but GT78 members with very high similarity to the bacterial mannosylglycerate synthase are present in *S. moellendorffiii*, *P. patens* (Table S1) and in the Charophycean green alga *Coleochaete orbicularis* (data not shown).

The majority of the enzymes required for building the vascular plant cell wall are still unknown and hence it is not possible to focus this study on the precise set of CAZy-families required for cell wall synthesis. A global approach has been taken, but families known to be involved in starch biosynthesis, for example, are not included in our analysis with its focus on cell wall biosynthesis. Evidence for involvement in cell wall synthesis in the remaining families is often quite circumstantial. For example, in GT64 loss-of-function of one Arabidopsis member (EPC1, At3g55830) has been shown to perturb the cell wall [20], [21], but the evidence for a direct involvement of EPC1 in cell wall biosynthesis is not clear. GT64 is divided into three groups with *S. moellendorffii* members in all three (Figure S1).

### Glycosyltransferases involved in cell wall biosynthesis

#### The cellulose synthase superfamily

The CAZy GT2 family is very large, including many enzymes not found in plants such as chitin synthase, hyaluronan synthase and others involved in protein N-glycosylation such as dolichyl phosphate β-glycosyl transferase. We have limited our analysis to the *CELLULOSE SYNTHASE* (*CESA*) superfamily, which includes proteins known and proposed to be involved in plant cell wall biosynthesis. The *CES* superfamily took its name from the *CESA* genes, which encode the catalytic subunits of cellulose synthase, and includes nine families of *CELLULOSE SYNTHASE-LIKE* (*CSL*) genes proposed to encode the GTs that synthesize the backbones of other cell wall polysaccharides [22]. The CESAs and CSLs are integral membrane proteins that are active in the plasma membrane (CESAs) or Golgi (at least some CSLs). Members of the *S. moellendorffii* CESA superfamily have been named using the established convention [22]. For historical reasons, we have included the callose synthases from the GT48 family in this section. Based on the ubiquitous presence of callose in *in vitro* cellulose biosynthesis assays, it was once hypothesized that cellulose and callose were synthesized by the same enzyme [23], [24]. However, it was subsequently discovered that callose synthases are similar to the yeast b-1,3-glucan synthase Fks [25], which is a member of GT48. All members of family GT48 are expected to be b-1,3-glucan synthases so no clade names are proposed for GT48 (Figure S2).

Analysis of the *CESA* gene superfamily in *S. moellendorffii* revealed many similarities with that of *P. patens*, including 1) absence of orthologs of the spermatophyte *CESA* genes that are specialized for primary and secondary cell wall biosynthesis, 2) presence of genes with similarity to cyanobacterial and red algal *CESA* genes, which are absent in spermatophytes, and 3) presence of only three *CSL* gene families, compared to seven or eight in seed plants (Figures 4, S3 and S4). The *CESA*, *CSLA*, *CSLC* and *CSLD* gene families of *S. moellendorffii* are smaller than those of either *P. patens* or seed plants and phylogenetic analysis revealed that each of these families diversified independently within the bryopsid, lycopod and seed plant lineages.

In Arabidopsis, the proteins encoded by three *CESA* genes (AtCESA4, 7, 8) are required for secondary cell wall biosynthesis in vascular tissue [26]. Proteins encoded by three different *CESA* genes (AtCESA1, 3 and either AtCESA2, 5, 6 or 9) are required for primary cell wall biosynthesis [27], [28]. All spermatophytes examined to date have orthologs of at least three primary wall and three secondary wall Arabidopsis *CESA* genes [29], [30]. *P. patens* lacks orthologs of these specialized *CESA* genes, as might be expected for a non-vascular plant [31]. Surprisingly, these orthologs are also lacking in *S. moellendorffii* (Figure S2) indicating that *CESA* diversification and specialization for primary and secondary cell wall biosynthesis was not necessary for the evolution of vascular tissue. This observation and the observation that monolignol biosynthesis, at least partly, evolved convergently with euphyllophytes monolignol biosynthesis lend genomic support to structural observations suggesting that the vascular tissue in *S. moellendorffii* is not homologous to euphyllophytes vascular tissue [32], [33]. Analysis of *CESA* genes from basal euphyllophytes will be required to better understand the relationship between tracheary element/secondary cell wall evolution and *CESA* diversification.

**Figure 6:**
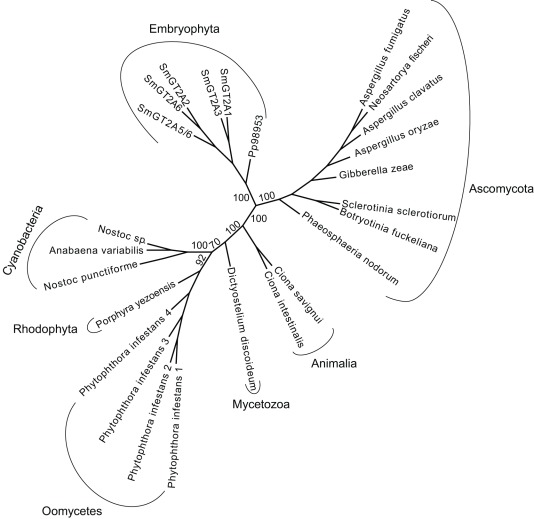
Phylogenetic tree of GT2. The genomes of *P. patens* and *S. moellendorffii* include GT2 sequences with no orthologs among seed plants. Putative cellulose synthases from cyanobacteria and the red alga *Porphyra yezoensis* are the most similar sequences from within the green plant lineage. Other similar sequences include known cellulose synthases from oomycetes, the slime mold *Dictyostelium discoideum*, and two species of tunicates, e.g. sea squirts (animalia), and ascomycete sequences of unknown function.

The CESA proteins of cyanobacteria [34] and the red alga *Porphyra yezoensis* [35] lack three defined regions (i.e. Zn-binding domain, Plant Conserved Region, and Class Specific Region) that are conserved in streptophyte CESAs. Only the streptophyte-type CESAs are known to form rosette terminal complexes [36] and the *P. yezoensis* CESAs form linear terminal complexes [37]. In addition to seven streptophyte-type *CESA* sequences, *P. patens* have one gene that is similar to cyanobacterial and red algal *CESA*s and sequences from ascomycete fungi. Similar sequences are not found in spermatophytes, but occur in the genome sequence of *S. moellendorffii* where they exist as a tandem duplicate and a group of four tandem genes. Some of the genes in the tandem repeats are pseudogenes and one, GT2A5/6, appears to be a fusion between two genes. So five full length GT2A's can be identified in the genome of *S. moellendorffii* (Figure 6). Although similar to *CESA*s from early-divergent taxa, these GT2 genes have undergone substantial diversification in *S. moellendorffii*, perhaps reflecting acquisition of a novel function.

The *CESA* gene superfamily also includes *CSLA* genes, which encode (gluco)mannan synthases [38], [14]; *CSLC* genes, which putatively encode XyG synthases [39]; *CSLF* [40] and *CSLH* [41] genes, which encode mixed-linkage b-glucan synthases; and five additional families of *CSL* genes, which have been proposed to encode the synthases that polymerize other cell wall polysaccharides [22], [42]. Only the *CSLA*, *CSLC* and *CSLD* gene families are represented in the genomes of *P. patens* [31] and *S. moellendorffii* (Table S1). This indicates that the *CSLB*, *CSLE*, *CSLF*, *CSLG*, *CSLH* and *CSLJ* gene families, and presumably novel cell wall polysaccharides synthesized by their encoded proteins, originated after divergence of the lycopsids from the vascular plant lineage. This observations is quite enigmatic as the biochemical analysis of *S. moellendorffii* cell walls revealed the presence of MLG. In the Poales, MLG is a product of the GTs encoded by *CslFs* and *CslHs*, but orthologues of these genes are not found in *S. moellendorffii*. This implies that the MLG in *S. moellendorffii* is produced by a different biosynthetic route than in Poales species and hence has emerged at least twice in embryophytes by convergent evolution. This raises the question of which *S. moellendorffii* GTs are responsible for the production of MLG. No obvious candidates emerge from the GTs identified in this study, indicating they may not be included in the CAZy database. Phylogenetic analysis revealed independent diversification of the *CSLC* and *CSLD* (Figure S4 and S5) gene families within the moss and lycophyte lineages. Although a catalog of *CESA* superfamily genes from sequenced plant genomes reported larger numbers of *CESA* and *CSL* genes in *S. moellendorfii* [43], these include allelic variants and two pseudogenes.

Is CSL function conserved among the major land plant lineages? Mannan/glucomannan synthase activity has been demonstrated directly for *P. patens* CSLA proteins [14], indicating that CSLA function is conserved. In contrast, the role of CSLC proteins in the synthesis of the XyG backbone has been examined only in seed plants and only shown for one member of CSLC [39]. As shown in this and previous studies, both mannan and XyG polymers are present in *P. patens.* CSLD proteins are involved in tip growth and early cell differentiation [44], [45]. Similarity to CESAs and complementation experiments have led to suggestion that CSLDs may be glucan synthases [46], [47] but heterologous expression of CSLDs in tobacco indicated that they have mannan synthase activity [44].

#### Xyloglucan and galactomannan

The biosynthesis of XyG is well understood with members of the GT2 CSLC-clade, GT34, GT37 and GT47 families implicated. The Arabidopsis XyG xylosyltransferase activities (At3g62720 and At4g02500, [48]) are placed in clade A of family GT34 with one rice and the S. moellendorffii sequences GT34A1 and GT34A2 (Figure S6). Surprisingly, no P. patens sequences could be found in clade A. The ancestral nodes of GT34 are not resolved but clades, named A through to D, are distinguishable in the tree and supported by high bootstrap values. The Arabidopsis proteins in clade C may also be XyG xylosyltransferases [49], and that could suggest that the P. patens proteins in clade D are xyloglycan xylosyltransferase as well. A much more detailed study of the proteins in clades A, C, and D will be required to clarify the divergence of these enzymes, which may all catalyze the same reaction. P. patens entries are found in clade B, which includes galactomannan galactosyltransferases. The antibody BS-400-4 and LM21used to detect mannans in P. patens and S. moellendorffii, respectively, has a rather broad specificity, hence binds to both β-(1,4)-D-mannan and galacto-β-(1,4) -D-mannan [50], [51](Petersen HL and Willats WG, unpublished). The galactomannan structure has thus not been resolved in P. patens or S. moellendorffii, so the sequence analysis cannot be correlated with biochemical evidence.

**Figure 7:**
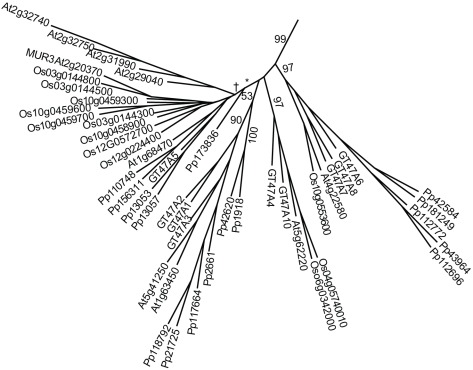
Phylogenetic tree of the A-clade of GT47, which includes the known XyG galactosyltransferase MUR3 and putative galactosyltransferases GT13 and GT18. The A-clade includes a mixed subclade with an overrepresentation of *P. patens* and *S. moellendorffii* sequences, a subclade containing At5g62220 with no *P. patens* members, and a subclade containing At5g41250 with no rice members, but is otherwise poorly resolved. Naming according to Li et al (2004): At5g62220 (GT18), At2g32740 (GT13) and At1g68470 (GT17). GT47 clade A members that are not included due to incomplete sequences include At4g13990, Pp156314 and Pp201625. Branching distal to † is not resolved.

Three genes, At2g20370 (MUR3), At2g32740 and At5g62220, proposed to be involved in galactosylation of XyG, are placed in clade A in GT47 [52], [53](Figure 7). Mur3 and At2g32740 are placed in a low-resolution subclade along with *S. moellendorffii*, *P. patens* and rice sequences (Figure 7). At5g62220 is, along with only *S. moellendorffii* (SmGT47A10 and SmGT47A4) and rice, placed in a resolved subtree (Figure 7). Which galactose in XyG that At5g62220 transfers is unknown. As there are no *P. patens* orthologs of At5g62220 it could be proposed that it transfers galactose to the ultimate α-1,6-xylose in the tetra or pentamer subunit in XyG as this galactose is substituted with galacturonic acid or arabinose in *P. patens* [12].

**Figure 8:**
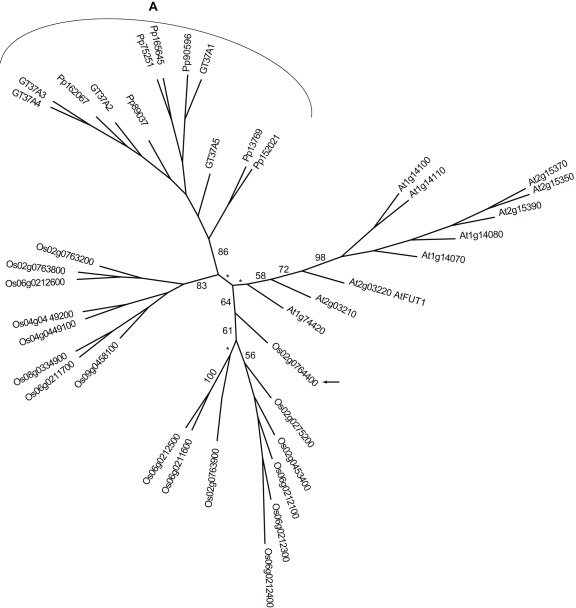
Phylogentic tree of GT37. The lower plant clade A is clearly resolved while the structure of the remainder of the tree is ambiguous. Known activity is xyloglucan α-1,2-fucosyltransferase AtFUT1. The other members of the Arabidopsis group are known not to fucosylate xyloglucan, but are likely to be fucosyltransferases (and are named AtFUT2-10). Os02g0764400 (arrow) is the most similar rice sequence to AtFUT1 although orthology is not clearly suggested by the clade structure. Adding *Brachypodium distachyon* GT37 sequences to the analysis (data not shown) will move Os02g0764400 and its *Brachypodium* ortholog into the putative Arabidopsis clade clearly showing that the clade structure is not stable. GT37 candidates that are not included due to incomplete sequences include At2g15350, Pp23092, Pp45180, Pp45146, Pp89038 and SmGT37A6.

The occurrence of fucose in XyG in different seed plant families has a bearing on the interpretation of the phylogentic relationships of family GT37 for *S. moellendorffii* (Figure 8). GT37 comprises several Arabidopsis fucosyltransferases, of which one and only one, AtFUT1, is a XyG fucosyltransferase [54]. Two other members of the GT37 FUT clade have been implicated in fucosylation of AGP [55]. Both, failure to detect terminal fucose by linkage analysis in rice and oat cell walls [56], [57], [58] and orthology of GT37-member Os02g0764400 with AtFUT4 and 5, which do not fucosylate XyG [59], supports the hypothesis that grass XyG is not fucosylated [60]. However, fucosylated XyG has been detected by radiolabeling in the grass *Festuca arundinacea* [61], so fucosylation persists at least in some genera of the grass family or possibly occurs transiently during biosynthesis as recently reported for xylosylation of XyG [62]. XyG of the moss *P. patens* is of the non-fucosylated type as judged by probing with monoclonal antibody CCRCM1, which recognize fucosylated XyG [11]. This antibody binds to *S. moellendorffii* cell wall extracts (Results not shown), and its GT37 members could include fucosyltransferases, but *S. moellendorffii* only contains GT37A members, a clade with only *P. patens* and *S. moellendorffii* GTs, and thus no ortholog of AtFUT1.

### Xylan

Xylans are the other major class of hemicellulosic polysaccharides and some progress has recently been made towards understanding its biosynthesis. The xylan backbones of several different species of gymnosperms and angiosperms have been shown to have a complex reducing end oligosaccharide hypothesized to be a primer or terminator for the synthesis [63]. Synthesis of the oligosaccharide in Arabidopsis is believed to involve IRX8 and PARVUS (GT8) and IRX7 (Figure S7) all of which appear to have orthologs in *S. moellendorffii* suggesting that it also makes the reducing end oligosaccharide. *S. moellendorffii* has only one member of the GATL group of GT8, to which PARVUS belongs (Figure S8). In contrast, Arabidopsis has 10 GATL members. Since the *parvus* mutant has a very strong phenotype, these 10 GATL members do not seem to be redundant [64]. The *S. moellendorffii* GATL is not a clear ortholog of PARVUS so we cannot safely conclude that it has the same function as PARVUS in xylan biosynthesis. The GTs responsible for elongation of the xylan backbone in Arabidopsis are likely to be IRX9/IRX9L and IRX14/IRX14L, which are located in different subgroups of GT43 (Figure S9)[65]. The close homologs IRX10 and IRX10L of GT47 are also involved in xylan backbone biosynthesis [66], [67]. Both *S. moellendorffii* and *P. patens* have IRX14/IRX14L and IRX10 orthologs. *S. moellendorffii* lacks direct orthologs of IRX9 but has orthologs of IRX9L (At1g27600). Both IRX9 and IRX14 are highly expressed in tissues containing secondary cell wall, but IRX14 follows this pattern less stringently [65], and the IRX9L gene in Arabidopsis is ubiquitous expressed according to *in silico* expression profiling [68]. Thus, it can be hypothesized that the seed plants have evolved a special isoform of IRX9, which is specific for secondary cell wall biosynthesis, while *S. moellendorffii* and *P. patens* only have the original isoform without a tissue specific role. This lack of GT specialization in *S. moellendorffii* is in line with the lack of specialized forms of CESA for secondary walls in this species (see discussion on cellulose synthase superfamily). The GTs, glucoronosyltransferases (GUX), that add glucoronic and methyl-glucoronic acid to xylan have been identified, [69], [70] as member of GT8 within a clade containing starch initiation proteins [71], [64]. Both *S. moellendorffii* and *P. Patens* have GUX orthologs but they are placed basal in the phylogenetic tree so possible specialization of GUX in higher plants has apparently not occurred in *P. patens* and *S. moellendorffii* (Figure S10). An alternative possibility is that all the higher plant GUX proteins have the same activity. GT61 is a family with many GTs in rice, several of which are coexpressed with genes predicted using *in silico* analysis to be involved in arabinoxylan biosynthesis [72], [70]. All four species have members in the A and B-clades of GT61 (Figure S11 and not shown). No activities are known for the A-clade whereas the distantly related B-clade contains the Arabidopsis β-1,2-xylosyltransferase involved in protein N-glycosylation and an apparent ortholog from each species [73]. The C and D clade of family GT61 is greatly expanded in grasses, with 18 rice entries compared to two for Arabidopsis and no members from either *P. patens* nor *S. moellendorffii*, suggesting that these enzymes catalyze a reaction that predominates in grasses. Mitchell et al. first proposed that the proteins could be responsible for β-1-2 xylose transfer onto arabinose sidechains of xylan [74], [72]. Recently an activity has been identified in GT61, a wheat clade C ortholog was shown to be a xylan arabinosyltransferase [75]. The LM10 antibody, which shows that xylan is present in *S. moellendorffii*, binds to unsubstituted or lowly substituted xylan. Determination of the fine structure of *S. moellendorffii* xylan would be helpful to the deductions of activities in GT61.

β-1,4-xylan is not known to be present in algae and based on the genetic complexity with the involvement of at least seven different activities for xylan biosynthesis, xylan biosynthesis is illustrative for the evolutionary jump that the earliest plants made in order to synthesize a cell wall suited for the terrestrial environment.

### Pectin

Pectin is the most complex cell wall polysaccharides, and their biosynthetic pathways are poorly understood [76], [77], [78], [79], [80]. The synthesis of the α-1,4-linked galacturonan backbone of homogalacturonan is catalyzed by GAUT members of family GT8, the galacturonosyl transferases. Presumably, the galacturonan backbones of RG-II and xylogalacturonan are synthesized by members of the GAUT family as well. Genes similar to the GAUT1 homogalacturonan synthase gene (At3g61130) are present in *S. moellendorffii* and *P. patens*, consistent with the presence of homogalacturonan in both species [81], [82]. The GAUT group within GT8 has subgroups that are missing in *S. moellendorffii* and *P. patens* or are missing only in *P. patens* (Figure S8). Of special interest is the clade containing AtGAUT7 (At2g38650), which has only angiosperm members. AtGAUT7 has been proposed to be part of a complex of GTs involved in homogalacturonan biosynthesis, even though homogalacturonan synthase activity has not been shown for AtGAUT7 [76]. Quasimodo1 (At3g25140) has been shown in Arabidopsis to be involved in homogalacturonan synthesis by mutation analysis [83]. The exact activity of Quasimodo1 has not been established and pleiotropic effects have been observed in the mutant [84]. *P. patens* does not have an ortholog of Quasimodo1 whereas *S. moellendorffii* does, pointing to a possible gradual evolution of homogalacturonan biosynthesis with complex formation requiring GAUT7 in angiosperms as the most recent development.

Family GT47 comprises, inter alia, GTs involved in pectin side-chain synthesis [85], [86]. A homolog of putative arabinan arabinosyltransferase ARAD1 was found in *S. moellendorffii* but not *P. patens* (Figure S7). As arabinan is found in *P. patens*, other members of the GT47B clade could be redundant to *ARAD1*, supported by the presence of residual arabinan in *arad1*. The arabidopsis xylosyltransferase XGD1 involved in xylogalacturonan biosynthesis lacks orthologs in both *P. patens* and *S. moellendorffii.* Interestingly, the most similar *S. moellendorffii* gene, GT47C1, occupies a separate subclade, which also has a *P. patens* member.

**Figure 9:**
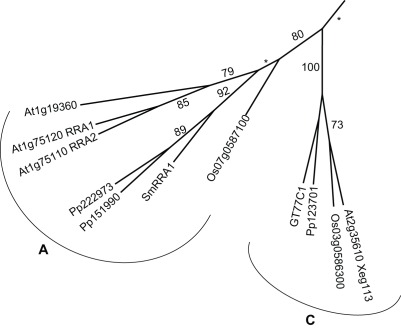
The A and C clades of GT77. The A clade holds the genes affected in the *reduced residual arabinose* (*rra*) mutants considered to be impaired in extensin arabinosylation. The arabidopsis member of the C-clade has recently also been implied in extensin arabinosylation based on analysis of the *xeg113*-mutant. Both clades have members from all four taxa. The full GT77 tree is Fig. S10.

Only one activity involved in RG-II biosynthesis has been identified, the RGXT xylosyltransferases of family GT77 [87], [88]. While Arabidopsis has three or four RGXT paralogs [87], [88] in the B-clade, rice, *P. patens* and *S. moellendorffii* have a single RGXT ortholog (Figure 9 and Figure S12). Although the complete RG-II molecule has never been detected in a moss, the presence of RGXT orthologs and finding of diagnostic sugars of RG-II yield evidence for presence of at least a precursor oligosaccharide homologous to RG-II [89].

### Extensins and AGPs

Extensins are structural wall polymers comprising a protein backbone that is glycosylated on contigous hydroproline residues [90]. The glycans are typically (β-1,2-Araf)_3_, where most side chains carry a terminal α-1,3-Araf. The RRACs in GT77A and Xeg113 of clade C are putative β-arabinosyltransferases transferring the second and third β-arabinose, respectively (Figure 9) [91], [92], [93]. Extensins are regarded as ancient because a quite similar group of proteins make up the wall of the chlorophyte alga *Chlamydomonas reinhardtii* albeit with a somewhat richer set of glycan structures [94] and both *P. patens* and *S. moellendorffii* have GT77 clade-A and C members.

AGPs are cell wall glycoproteins that are attached to the plasma membrane through glycosylphosphatidylinositol lipid anchors, reviewed recently and suggested clade naming convention is followed here [95], [96], [97]. It is generally assumed that the plant members of family GT31 comprise activities involved in adding galactosyl units to *O*-glycans of AGPs. This is based on the observations that animal GT31 all appear to transfer hexosyl monosaccharides in β-1,3 [98] and preliminary studies have shown galactosyltransferase activity for several Arabidopsis members of the B-clade of GT31 (N. Geshi, E. Knoch, and H.V. Scheller, unpublished) and one member of the C-clade (I. Damager, C. Toft Madsen, B. Petersen, J. Harholt and P. Ulvskov, unpublished). Only a single plant member of GT31, At1g26810 belonging to the B-clade, has been functionally characterized and shown to be a galactosyltransferase involved in the synthesis of the Fuc α-1,4 (Gal β-1,3) GlcNAc-R Lea structure of N-glycans [97]. At1g26810 belongs to the B clade of GT31 comprising a pfam 01762 galactosyltransferase domain, and a galactoside binding lectin domain pfam 00337, yet good evidence was presented that the B-clade sequences are not paralogs [97]. At1g26810 alone is implicated in Lea structure synthesis. This raises a problem in gene naming. The *S. moellendorffii* gene, prot ID = 183099, which is closest to At1g26810 is probably closer to At3g06440 known not to be involved in Lea synthesis. *S. moellendorffii* has orthologs to both the (α-1,3-FucTs) in GT10 and to FuctC (α-1,4-FucT) which takes part in Lea synthesis. This raises substantial doubt as to the presence of the Lea structure in *S. moellendorffii* and we have named the gene closest to AtGALT1 GT31B1 awaiting biochemical evidence (see Figure S13 and S14).

A large group of mammalian member s of GT31 are the fringe GTs, which are N-acetyl-glucosaminyltransferases. The fringe-related GTs in GT31 form clade C, which is distinct from clades B to F and is presented as a separate tree (Figure S15).

Two of the arabidopsis sequences outside CAZy identified as GT-candidates, At5g11730 and At3g21310, carried domain of unknown function DUF266 [99]. LOPIT proteomics experiments have localized At5g11730 to the Golgi [100]. Recently, DUF266s were found in a bioinformatics search for putative GTs in arabidopsis [101], and the brittle culm mutation *BC10* in rice was linked to a lesion in a gene with a DUF266-domain that encodes a GT potentially involved in AGP-biosynthesis [102]. BC10 is part of DUF266 Clade A comprising three subclades with unresolved common ancestry (Figure S16). BC10 has orthologs in both *S. moellendorffii* and *P. patens*. No CAZy family exists for the DUF266s yet due to the lack of biochemical evidence. Consequently, we do not propose names for the GTs at this point. The numbers in both the table and the tree are JGI protein IDs.

### Concluding remarks

Most of the cell wall polymers found in angiosperms are reported here to be present in both *S. moellendorffii* and *P. patens*. However, there were notable differences observed in the relative abundance of specific polymers and in some aspects of cell wall architecture. In contrast to most angiosperm cell walls, pectin appeared to be most abundant in the vascular tissue whilst xylan was observed mainly in cortical cell walls. Xylan and mannan appears to be the quantitatively most aboundant polymers, with minor amounts of pectin. MLG was also found in the cell wall of *S. moellendorffii*, intriguing since orthologues of neither *CslF* or *CslH* were identified in the genome of *S. moellendorffii*. These findings which suggest the evolution of diverse strategies for utilizing specific cell wall components may have some relevance with regard to the remodeling of cell wall structures that is required for optimizing plant biomass for biofuel use.

The comparative genomic approach used in the building of the *S. moellendorffii* CAZyome gave rise to interesting discussions but no clear functionality could be assigned, most likely due to the overlapping presence of cell wall polymers in the four species analyzed. We have presented a gene naming convention for GTs similar to that already in place for the model grass *Brachypodium disctachyon*. The next step would be to extend the analysis and include Charophycean and Chlorophycean algae. As an example of this were the cell wall related GT2 in some Chlorophycaen green algae analyzed [103]. Both CslA/C orthologs were found, in agreement with the presence of mannans, and no streptophyte-type CESA was identified. But additional knowledge could be gained on for example extensin biosynthesis if the analysis was broadened to include the whole CAZyome.

## Materials and Methods

### Plant material

*Selaginella moellendorffii* for CoMPP and microscopy was obtained from Plant Delights Nursery, Inc., Raleigh, NC, USA and maintained under humid conditions with low light intensity. Alcohol insoluble residue (AIR) was made by freezing tissue in liquid nitrogen and homogenizing using a TissueLyser (Sigma-Aldrich). Five volumes of 70% EtOH was added to the fine powder and incubated with rotation for 1 h. The material was spun down and the EtOH changed before another hour of incubation. This procedure was repeated five times before a final wash with acetone, and the samples were left to air dry.

### CoMPP

CoMPP was carried out essentially as previously described [11]. Cell wall polymers were sequentially extracted from 10 mg of AIR with 50 mM diamino-cyclo-hexane-tetra-acetic acid (CDTA), pH 7.5, and 4 M NaOH with 0.1% v/v NaBH_4_, and extractions printed in three dilutions and three replicates giving a total of 9 spots per sample. The entire experiment was repeated three times and the average of these three experiments is reported. The data were converted into a heatmap format using the online BAR heatmapper tool (http://bar.utoronto.ca/ntools/cgi-bin/ntools_heatmapper.cgi). All JIM, LM and Mac antibodies as well as CBM3a were obtained from PlantProbes, UK, and BS 400-2 and -4 was obtained from Biosupplies, Australia. Secondary alkaline phosphatase anti-Rat and anti-Mouse antibodies were obtained from Sigma-Aldrich.

### Fluorescence microscopy

Transverse sections of fresh *S. moellendorffii* stems were cut using a vibrating knife microtome (Vibratome, St. Louis, MO 63134, USA). Sections were incubated for 1 h in primary antibody diluted 1∶10 in phosphate buffered saline (PBS, 140 mM NaCl, 2.7 mM KCl, 10 mM Na_2_HPO_4_, 1.7 mM KH_2_PO_4_, pH 7.2) containing 5% (w/v) fat-free milk powder (MP/PBS). mAbs were used with specificity for HG (JIM5) [104], α-(1,4) xylan (LM10)[105], β-(1,4)-galactan (LM5)[106] and α-(1,5)-arabinan (LM6)[107]. After washing in PBS, sections were incubated for 1 h in fluorescein isothiocyanate (FITC)-conjugated secondary antibody (anti-rat-FITC, Sigma, Poole, UK). After washing in PBS, sections were mounted in antifade agent (CitiFluor, Leicester, UK) and examined using a laser scanning confocal microscope (LSCM510, Carl Zeiss, Switzerland).

### Sequential extraction and sugar composition analysis

AIR was sequentially extracted with 50 mM diamino-cyclo-hexane-tetra-acetic acid (CDTA), pH 7.5, and 4 M NaOH with 0.1% v/v NaBH_4_. Approximately 30 mg AIR was extracted with 1 ml of solvent. Both extractions were carried out at room temperature for 24 hours with 1400 rpm shaking. AIR, residue and extractions were hydrolysed with triflouroacetic acid and released sugars were detected and quantified as previously described [108].

### Proteomes and database creation

The filtered models proteome of *S. moellendorffii* was acquired from the JGI web-site. Protein sequences of the CAZy-database were downloaded from NCBI and used for generating a CAZy– blast database. The Arabidopsis proteome was downloaded from TAIR and all genes already in CAZy were subtracted and an Arabidopsis blast database depleted of known GTs was prepared.

### The screen

The *S. moellendorffii* proteome was blasted against both the CAZy-blast-database and the GT-depleted arabidopsis database using an e-value of 10^−25^ as threshold. Hits to CAZy for which a better (e-value smaller by a factor of five or more) hit was found in the GT-depleted Arabidopsis database were eliminated. The remaining candidates were then screened by an rpsblast against the conserved domain database CDD [109]. Both rpsblast and the CDD are made available by NCBI. CDD does not cover all families, for those families not covered, fold prediction by the Phyre-server was employed [110]. We have attempted not to give names to obvious fragments and pseudogenes, so the final threshold to pass was based on manual screening of alignments of *S. moellendorffii*, Arabidopsis, rice and *P. patens* sequences in the given family. Erroneous *in silico* sequence predictions, mostly wrongly predicted start site and intron/exon borders, were manually edited and corrections were uploaded to JGI. New protein ID number was assigned when editing so please refer to Table S1 for file containing all corrected protein IDs used in present study. Uncorrected sequences can still be obtained by BLAST on JGI's Selaginella site (http://genome.jgi-psf.org/cgi-bin/runAlignment?db  =  Selmo1&advanced  = 1).

The GT2 and GT8 families were handled individually. GT8 is a very divergent family and the analysis was limited to the GAUT, GATL and GUX groups of genes. Likewise analysis of the very large GT2 family was limited to identification of members of the plant CESA superfamily and genes with similarity to cyanobacterial and algal cellulose synthase genes. Representative members of the CESA and CSL families from Arabidopsis, rice and *P. patens* and cyanobacterial, green algal and red algal cellulose synthases were used to search the *S. moellendorffii* genome with the internal tblastn function. All hits were examined and gene models were identified and edited manually.

The proposed gene names were used for the annotation in JGI's database and may be searched at http://genome.jgi-psf.org/annotator/servlet/jgi.annotation.Annotation?pDb  =  Selmo1

### Phylogenetic analysis

Sequences were aligned using ClustalX 2.0 with standard settings and applying the Gonnet substitution matrix series. Areas with poor resolution were deleted using Bioedit [111] and sequences were realigned. Several rounds of editing were done if needed. *P. patens*, rice or Arabidopsis sequences with obvious and large mistakes, be it annotation mistakes or pseudogenes, were not included in the trees, but protein ID are included in figure legends. Phylogenetic trees were generated by maximum likelihood using proML from the phylip package v3.68 [112] with 250 bootstraps from seqboot, using Jones-Taylor-Thornton matrix [113], no global rearrangement, rough analysis and one jumble. For clarification, cosmetic rearrangement of the trees was made using Adobe Illustrator (Adobe, USA.

## Supporting Information

Figure S1 **Phylogenetic tree of GT64.** The family is not well characterized. Analysis of *epc1* (At3g55830) from clade A indicated that GT64 could be involved in cell wall biosynthesis. No putative function for the GT64A or the uncharacterized GT64B and C has been reported.
(EPS)

Figure S2 **Phylogenetic tree of GT48.** This family includes the callose synthases abbreviated GSL for glucan synthase like. No other activities are known from GT48 and the *S. moellendorffii* members are distributed throughout the tree. GT48 candidates that are not included due to incomplete sequences include At2g36850 and At5g36810.
(EPS)

Figure S3 **Phylogenetic tree of the** ***CESA*** **family.** Arabidopsis genes required for synthesis of cellulose in primary (subgroups P1, P2, P3) and secondary (subgroups S1, S2, S3) cell walls have orthologs in rice and other seed plants. *S. moellendorffii* and *P. patens* CesAs form species-specific subgroups and lack orthologs of seed plant CesAs specialized for primary and secondary cell wall synthesis. The two *P. patens* genes marked with a * (PpCesA6 and PpCesA7) do not have JGI protein ID numbers because they failed to assemble completely due to their extremely high similarity. They are designated by the GenBank accession numbers for their full-length cDNA sequences. SmCESA5 is not included in the tree.
(EPS)

Figure S4 **Phylogenetic tree of the CslA family.** Species-specific subgroups are well supported. Mannan synthase and glucomannan synthase activity has been demonstrated for proteins encoded by AtCslA1,2,3,7,9 and PpCslA1 (183385) and 2 (179490). GT2 CslC. A mixed Arabidopsis and rice subgroup and a rice subgroup are well supported. Heterologous expression of AtCslC4, a member of the mixed subgroup, resulted in production of b-1,4-glucan. Members not included in tree comprise Os07g0124750 and Os07g0630900.
(EPS)

Figure S5 **Phylogenetic tree of the CslD family.** Arabidopis genes with phenotypes in root hairs (AtCslD 2,3), pollen tubes (AtCslD 1,4), and stems (AtCslD5) all have rice orthologs. *S. moellendorffii and P. patens* CslDs form species-specific subgroups.
(EPS)

Figure S6 **Phylogenetic tree of GT34.** Known activities, xyloglucan α-1,6-xylosyltransferases, AtXXT1 and AtXXT2 are found in clade A while AtXXT5 is in clade C. Arrows mark the likely Arabidopsis orthologs of the *Cyamopsis* galactomannan α-1,6-galactosyltransferase. The large B-clade comprises species-specific subgroups, but the basal topology is not resolved.
(EPS)

Figure S7 **Phylogenetic tree of GT47.** The family includes inverting activities using a range of donors and acceptors. Naming of this family was established by Li et al., (2004). Subclade A contains xyloglucan galactosyltransferases (see also Figure 6), subclade B includes putative arabinan arabinosyltransferases, subclade C contains at least a xylogalacturonan xylosyltransferase (and probably other activities), subclade D contains members with unknown activity putatively involved in xylan biosynthesis (subgroup neighbouring to subclade E) and putatively involved in biosynthesis of the reducing end tetrasaccharide sequence of xylan (subgroup neighbouring to subclade F). Nothing is known about the activities present in subclade E or F. Candidates not included in tree comprise Os03g0182300, Os04g0633450, Os06g0176100, Pp156314, Pp201625, Pp214811, At4g13990, At5g37000.
(EPS)

Figure S8 **Phylogenetic tree of GT8.** The GAUT and GATL clades of GT8 are presented here. The other members of GT8 are too divergent from the GAUT and GATL clades to be included in a single tree. The GAUT clade includes a homogalacturonan synthase (At3g61130, AtGAUT1) and an enzyme involved in homogalacturonan biosynthesis putatively a homogalacturonan synthase (At3g25140, AtQUA1). The GATL clade is putatively involved in biosynthesis of the reducing end tetrasaccharide sequence of xylans based on knock out mutant analysis of PARVUS (At1g19300). Candidates not included in tree comprise Pp123164 and Os10g0454100.
(EPS)

Figure S9 **Phylogenetic tree of GT43.** The family is divided into two subclades, both of which are likely to contain xylan backbone xylosyltransferases based on analysis of Arabidopsis mutants.
(EPS)

Figure S10 **Phylogenetic tree of the GT8 GUX clade.** This clade has been shown to contain xylan glucuronosyltransfereases.
(EPS)

Figure S11 **Phylogenetic tree of GT61.** The family includes arabinoxylan arabinosyltransferases (Clade C). Beside this activity, AtXYLT, a β-1,2-xylosyltransferase involved in protein *N*-glycosylation is also found in GT61. It belongs to a clade of GT61 (Clade B), which is not presented in the tree due to low similarity to the A and C clades. Arrows indicate those among the rice sequences that in particular drive the grass bias of the C-clade in the Mitchell et al. (2007) study. Os06g0707000 is not included.
(EPS)

Figure S12 **Phylogenetic tree of GT77.** Known activities include the RG-II α-1,3-xylosyltransferases RGXT1, RGXT2 and RGXT3. β-arabinosyltransferase activity is proposed for RRA1, RRA2 and Xeg113. Resolution in the part of the tree not assigned clade letter(s) is quite dependent on which species are included (data not shown). At5g40900 is in GT77 according to CAZy but has been excluded.
(EPS)

Figure S13 **Phylogenetic tree of GT10.** The family includes fucosyltransferases involved in *N*-linked glycosylation of proteins. The FucTC clade includes α-1,4 fucosyltransferases involved in biosynthesis of the Lewis glycan structure. The FucTA and FucTB clades include α-1,3 fucosyltransferases involved in biosynthesis of complex *N*-linked glycans.
(EPS)

Figure S14 **Phylogenetic tree of clade B of GT31.** A galactosyltransferase activity involved in biosynthesis of the *N*-linked glucan (AtGalT1) is the only known activity. Two other subclades contain only flowering plant sequences.
(EPS)

Figure S15 **Phylogenetic tree of c-clade of GT31.** The clade consists of two well resolved subclades with only flowering plant sequences and a subclade containing also *P. patens* and *S. moellendorffii* sequences. The basal topology is not resolved. No evidence for the function of GT31C has been published. At5g57500 is not included.
(EPS)

Figure S16 **Phylogenetic tree of the DUF266 family.** This family is not presently recognized as a GT family but is likely to be included in CAZy as bioinformatic approaches and mutant studies have implicated the DUF266 proteins as GTs. Three clades are recognised. Members of the A clade have been proposed to be involved in AGP biosynthesis based on analysis of the *osbc10* mutant. At4g31350 is not included.
(EPS)

Table S1 ***S. moellendorffii*** **and** ***P. patens*** **protein IDs and gene names listed by family and clade.**
(XLS)
